# Male-killing bacteria in insects: mechanisms, incidence, and implications.

**DOI:** 10.3201/eid0604.000402

**Published:** 2000

**Authors:** G D Hurst, F M Jiggins

**Affiliations:** Department of Biology, University College London, United Kingdom. g.hurst@galton.ucl.ac.uk

## Abstract

Bacteria that are vertically transmitted through female hosts and kill male hosts that inherit them were first recorded in insects during the 1950s. Recent studies have shown these "male-killers" to be diverse and have led to a reappraisal of the biology of many groups of bacteria. Rickettsia, for instance, have been regarded as human pathogens transmitted by arthropods. The finding of a male-killing Rickettsia obligately associated with an insect suggests that the genus' members may be primarily associated with arthropods and are only sometimes pathogens of vertebrates. We examined both how killing of male hosts affects the dynamics of inherited bacteria and how male-killing bacteria affect their host populations. Finally, we assessed the potential use of these microorganisms in the control of insect populations.


### Male-Killing Bacteria in Insects: Mechanisms, Incidence, and Implications

**Gregory D.D. Hurst and Francis M. Jiggins**
University College London, United Kingdom

Bacteria that are vertically transmitted through female hosts and kill male hosts that inherit them were first recorded in insects during the 1950s. Recent studies have shown these "male-killers" to be diverse and have led to a reappraisal of the biology of many groups of bacteria. *Rickettsia*, for instance, have been regarded as human pathogens transmitted by arthropods. The finding of a male-killing *Rickettsia* obligately associated with an insect suggests that the genus' members may be primarily associated with arthropods and are only sometimes pathogens of vertebrates. We examined both how killing of male hosts affects the dynamics of inherited bacteria and how male-killing bacteria affect their host populations. Finally, we assessed the potential use of these microorganisms in the control of insect populations.

Female insects commonly interact with bacteria they pass on to their progeny. These inherited bacteria are often beneficial symbionts that play a key role in host metabolism. In many cases (e.g., the aphid symbiont *Buchnera*), the bacteria are maintained in a special host organ, the bacteriome, with the host controlling transmission to progeny, and show evidence of cospeciation (1,2). In these cases, destroying the bacteria (e.g., through antibiotic treatment) causes a profound loss of host performance. In other cases, inherited bacteria are not integrated into host physiology and anatomy and do not show long-lived relationships with their host, as indicated by a lack of cospeciation (3). These bacteria may be broadly separated into two classes. First, bacteria maintained through a phase of horizontal transmission (e.g., *Rickettsia prowazekii*), with transmission to other arthropod hosts often occurring through a vertebrate or plant intermediate host (infection of the intermediate host and new acquisition of infection follow from host feeding); second, bacteria that rarely show horizontal transmission, but are maintained because they manipulate host reproduction. One set of manipulations manifested by these bacteria is increasing investment in daughters at the expense of sons. In these cases, particular host lines produce female-biased sex ratios, a trait that is inherited but curable with antibiotics. We considered one class of these, the male-killing bacteria, in which infection of a female results in the production of female-biased broods because male progeny die during embryogenesis.

Address for correspondence: Department of Biology, University College London, 4 Stephenson Way, London NW1 2HE, U.K.; Fax: +44 20 73832048; e-mail: g.hurst@galton.ucl.ac.uk.

### Systematics of Male-Killing Bacteria

Molecular systematic approaches have shown that male-killing bacteria derive from many different clades. In most cases, the data come from DNA sequencing of bacteria associated with the trait and confirmation of the trait association by polymerase chain reaction across infected and uninfected lines. Because inherited microorganisms are difficult to culture, Koch's postulates have been fulfilled formally in only two cases (4,5). Given this caveat, male-killing bacteria have been found within the genus *Spiroplasma* (Mollicutes) (4,6), the Flavobacteria-Bacteroides group (7), and the gamma and alpha subdivisions of the proteobacteria (5,8,9) (Figure).

Male-killing bacteria derive from arthropod-associated bacterial clades that are not themselves male-killers. The clades can be separated into two types according to the transmission mechanisms of bacteria within them: first, entirely horizontal transmission or a mix of horizontal and vertical transmission; and second,

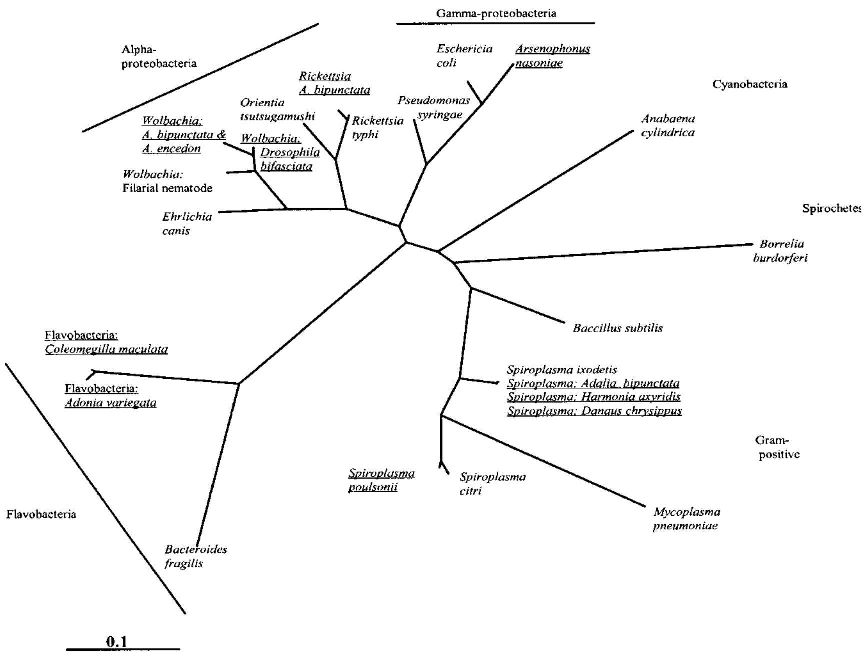

Figure. Phylogenetic relationship of male-killers and a selection of other eubacteria inferred from 16S rDNA sequences, using maximum likelihood implemented on PAUP*. The male-killing bacteria (underlined) have been labeled with the name of their insect host if a species name is not available. The relationships of the major bacterial groups are uncertain.

horizontal transmission that is not epidemiologically important. In the first type of clade are the genera *Spiroplasma* and *Rickettsia*. In *Spiroplasma*, most members have either horizontal transmission only (after feeding on a plant host) or a mix of horizontal and vertical transmission between arthropod hosts (10). *Rickettsia* most commonly have a mix of horizontal and vertical transmission, with horizontal transmission occurring after feeding on a vertebrate host. As recently as 10 years ago, *Rickettsia* was regarded as one of the vertebrate pathogens borne by arthropods. However, *Rickettsia* that show transmission after feeding on plant hosts are increasingly being recognized (11), and the finding of a male-killing *Rickettsia* in ladybird beetles (8) suggests that the group is associated with arthropods, some members of which cause disease in vertebrates. Other male-killing strains of *Rickettsia* will most likely be found. However, whether a bacterium from these groups could evolve male-killing yet retain horizontal transmission between females via feeding on a plant or vertebrate host has not been established. The fact that male-killers derive from such groups suggests this possibility.

In the second type of clade, vertical transmission rates far exceed those of horizontal transmission. *Wolbachia* and the flavobacterial lineage associated with arthropods are in this group. *Wolbachia* are usually maintained through manipulation of their host's reproduction (12). The closest relative of the flavobacterial male-killer is *Blattabacterium*, the beneficial inherited bacterium of cockroaches and termites (13).

Male-killing, a trait that evolves in bacteria already maternally inherited in arthropods, can occur if the ancestral agent is obligately vertically transmitted or a mix of vertical and horizontal transmission is present. Therefore, male-killing strains are likely to be common in the genus *Spiroplasma* and the alpha group of proteobacteria. Furthermore, the diversity of agents suggests that there is no taxonomic bar to where the transition to male-killing can take place. Thus male-killing strains are also likely to be found in the spirochetes and perhaps the beta and delta divisions of the proteobacteria, as these groups are known to be vertically transmitted within arthropods.

Although vertical transmission of male-killing bacteria is the rule, transmission between host species has occurred. In *Spiroplasma*, the relatives of *S. ixodetis* cause male-killing in distantly related hosts (a butterfly and a ladybird beetle) (6,14). The evolutionary distance between beetles and butterflies indicates that the bacteria do cross between host species over evolutionary time.

### Host Species Affected

The incidence of male-killing bacteria varies with host ecology and biology. The trait of male-killing is adaptive when the death of males promotes the survival of female siblings. If the bacteria can be transmitted only vertically, the death of male hosts can at worst be neutral (i.e., they cannot transmit the bacterium). Death of males is adaptive if it increases the survival of sibling females, who bear the same bacterium by virtue of common descent.

The features of host biology and ecology that increase the benefit to the bacterium of killing male embryos are sibling egg consumption (females eat their dead brothers), antagonistic interactions between siblings (male-killing may reduce both cannibalism of females and the intensity of competition between siblings), and deleterious inbreeding (15-17). These observations explain why male-killer hosts commonly lay eggs in clutches. Incidence is highest where there is also sibling egg consumption, as with coccinellid (ladybird) beetles. Approximately half of aphidophagous species bear male-killers, and one species (*Adalia bipunctata*) is host to at least three male-killing bacteria (6,8,9).

Male-killing bacteria have been recorded only in insects. However, the range of insect hosts is wide, with a variety of different sex determination systems. Given that close relatives of male-killing bacteria are found in noninsect arthropods (e.g., *Spiroplasma* and *Rickettsia* in ticks) and the conditions for the spread of male-killing strains are met outside insect hosts, cases of male-killing are likely to occur in species other than insects. Two examples merit particular examination. First, infection with *Orientia tsutsugamushi* is associated with production of all-female broods in the trombiculid mite, *Leptotrombidium fletcheri* (18,19); in this example, the nature of the resultant sex-ratio distortion (primary vs. secondary bias) needs to be assessed. Second, in the case of *Spiroplasma ixodetis* and its tick host *Ixodes pacificus*, the association of closely related bacteria with male-killing in insects needs to be assessed.

### Prevalence of Male-Killers in Natural Populations

The prevalence of male-killers in natural populations varies with host species (Table 1). A prevalence value of 5%-50% might be “normal” among female hosts; however, in some cases prevalence is very low (e.g., 1% in *Drosophila*

Table 1. Prevalence of male-killers in natural populations of their insect hosts (proportion of females infected)

| Bacterium | Host | Prevalence (%) | Ref. |
|---|---|---|---|
| *Spiroplasma* sp. | *Adalia bipunctata* | 0-22 | 6 |
| (*S. ixodetis* relative) | *Harmonia axyridis* | 0-49 | 35 |
| | *Danaus chrysippus* | 40 | 14 |
| *Spiroplasma poulsonii* | *Drosophila willistoni* group flies | 0-3 | 20 |
| *Wolbachia* | *Acraea encedon* | 61-95 | 36 |
| | *Acraea encedana* | 95 | 21 |
| | *Adalia bipunctata* | 0-5 | 9 |
| Unnamed Flavobacteria | *Coleomegilla maculata* | 23 | 37 |
| | *Adonia variegata* | 13 | 38 |
| *Arsenophonus nasoniae* | *Nasonia vitripennis* | 4 | 15 |
| *Rickettsia* | *Adalia bipunctata* | 5-7 | 6,39 |
| Unknown | *D. bifasciata* | 0-7 | 26 |
| | *D. prosaltans* | 13 | 33 |
| | *Gastrolina depressa* | 0-81 | 22 |
| | *Epiphyas postvittana* | 4-7 | 40 |
| | *Hypolimnas bolina* | 0-61 | 41 |
| | *Spodoptera littoralis* | 24 | 42 |
| | *Lymantria dispar* | 9 | 43 |

*willistoni* [20]), and in some exceptional species > 90% of females are infected (e.g., the butterfly *Acraea encedana* [21]). However, there is likely to be study bias towards high-prevalence infections, and all very low-prevalence infections occur in drosophilids, where large samples can easily be bred. Infection prevalence also commonly varies between populations within a host, and prevalence can vary on a remarkably small scale. In the walnut leaf beetle (*Gastrolina depressa*) in Japan, male-killers are absent in populations at the north and south of the islands but present in 50%-80% of females in the center of the islands (22). Prevalence variation on a kilometer scale exists in *Acraea encedon* (21).

Prevalence is determined by the physiologic effect of infection on female host performance, the transmission efficiency of the bacterium from mother to progeny, and the level of advantage to male-killing (determined by host factors such as sibling egg consumption) (Table 2). Transmission efficiency may be influenced by the environment (e.g., high temperatures may lower transmission efficiency), the bacterium, and the host. Selection favors host genes that impede the transmission of the bacteria from mother to progeny. The spread of host resistance genes may prevent infections from commonly reaching the high prevalence achieved by other inherited bacteria.

Table 2. Factors affecting the prevalence of male-killing bacteria

| |
|---|
| **Increase** |
| Decreased rate of inbreeding suffered by female hosts |
| Increased access to early resources through consumption of dead sibling male hosts |
| Increased access to resources due to reduced competition, following death of sibling male hosts |
| Direct physiologic benefits of infection |
| **Decrease** |
| Inefficiency in vertical transmission |
| Direct physiologic costs of infection |
| Local extinction of groups having a high prevalence of male-killers |

### Mechanism of Male-Killing

Little is known about how male-killing is achieved. Neither the cue used to detect sex nor the mechanism by which death is brought about is known in any detail. Indeed, rather than two steps (detection then virulence) there may be only one (constitutive production of a factor that causes death in males only). What we know derives almost exclusively from study of the interaction between *Spiroplasma poulsonii* with *Drosophila*.

Studies of embryos from *D. willistoni* lines infected with *S. poulsonii* show that death occurs at two stages (23): 1) before gastrulation, associated with abnormal cleavage patterns; in particular, achromatic spindles, with other abnormalities of the mitotic process, which account for most embryonic deaths in male-killed lines. 2) After gastrulation, not associated with the normal brown coloration of necrotic embryos; rather, the embryo blackens as a result of breakdown of internal structures and pycnosis of nuclei.

The points of interaction between host and bacterium have been investigated in *D. melanogaster* lines transfected with *S. poulsonii*. In *Drosophila*, sex is determined by the ratio of the X chromosomes to autosomes. In females, which are 2X:2n, the peptide Sxl is produced. Sxl induces female development of the soma and the germ line. In males, which are X: 2n, Sxl is not produced. Absence of Sxl is associated with upregulation of genes on the single X chromosome (dosage compensation), male somatic development, and male germ line development. In *Drosophila*, the male-killer does not interact with any part of the somatic sex development pathway. Mutants of the *tra* gene bear two X chromosomes and produce Sxl but develop as somatic males. They are not, however, killed by *S. poulsonii* (24). Thus, the interaction between male-killer and host is not associated with somatic sex, so the target of detection and virulence is either before Sxl is produced, Sxl itself, or the dosage compensation or germ-line determination pathways.

Although the interaction between *Drosophila* and *S. poulsonii* is the only one studied in any detail, it appears that the mechanism of sex determination exhibited by different male-killer hosts varies widely. Male-killing bacteria have been observed in male heterogametic, female heterogametic, and haplodiploid hosts. Furthermore, members of the same clade of male-killers can be found in hosts of different sex determination systems. The same *Spiroplasma* kills males in ladybirds (male heterogametic) and butterflies (male homogametic). Similarly, male-killing *Wolbachia* have been observed in both male and female heterogametic species

(9). Given that male and female heterogametic systems count chromosomes in opposite directions and show different patterns of dosage compensation, the fact that male-killers operate in both these hosts suggests that the X:autosome counting mechanism and the dosage compensation pathway may not be the focus of male-killing activity; rather, somatic sex determination or germ-line sex determination may be the focus.

Experiments with *S. poulsonii* demonstrate that the somatic sex determination system is not the focus of male-killing behavior. In the case of the other male-killing *Spiroplasma*, the presence of the bacterium in species of different sex determination systems suggests that the focus is either the somatic sex determination or the germ-line determination system. Two conclusions are therefore possible: germ-line determination is the focus of male-killing in all cases, or male-killing has more than one basic mechanism. Further research is clearly warranted.

### Direct Effects on Female Hosts

The interaction between male-killing bacteria and their female hosts is interesting. On the one hand, there is selection for a reduction in the number of bacteria present in the host (minimizing virulence) and for a direct physiologic contribution to host metabolism. On the other hand, their fitness is also associated with the fidelity of their transmission to progeny. There may be a trade-off between minimizing virulence and maximizing vertical transmission efficiency, especially if such efficiency is positively related to bacterial number. Thus these bacteria can be either detrimental (if the density of bacteria is high to ensure vertical transmission) or beneficial to the host (if the bacteria play a role in host metabolism).

Empiric studies have suggested that infection usually decreases the performance of female hosts (25,26). The one exception is the interaction between *Spiroplasma poulsonii* and members of the *Drosophila willistoni* group, in which larval development is accelerated by infection (27,28). However, infection is also associated with increased sterility and decreased longevity among adult females (28). Male-killing bacteria, unlike beneficial symbionts, are spread throughout host tissues, and the bacteria may be present in very high numbers. *Drosophila* are infected with extremely high titers of *S. poulsonii* within the hemolymph (29). *Adalia bipunctata* hemocytes are regularly infected with *Rickettsia* (30).

Beneficial effects of male-killing bacteria on host performance cannot yet be ruled out. However, positive effects may be fewer than those found in the "classical" beneficial agents, which typically perform a vital metabolic function that insects are unable to perform. Male-killers infect a minority of females and are rarely carried by larval or adult males. Thus, although they may add to host performance, they cannot substitute for any part of it. A host cannot be dependent on a male-killer for a physiologic function as it can on a beneficial symbiont.

### Population and Evolutionary Effects on Hosts

Invasion of a host population by male-killing bacteria affects the dynamics of the host population and alters the pattern of selection on the population to ameliorate the effects of the parasite (Table 3). A high prevalence of male-killers may increase the proportion of female hosts that fail to mate (31), potentially reducing the population size of the host. A dearth of males can subtly alter the mating system of the host. Choice by females of male mates and competition among males for mating opportunities are the

Table 3. Population and evolutionary effects of invasion of a host by male-killing bacteria

| Effects on population level | Evolutionary effects |
|---|---|
| Reduced population density at larval level due to death of male embryos | Selection for increased host clutch size |
| Failure of females to find mates where parasite prevalence leads to shortage of males, with potential effects on adult population size | Selection for genes that prevent transmission or action of male-killer |
| Altered epidemiology of sexually transmitted pathogens due to increased reproductive success of males | Alteration in host pattern of sexual selection due to alteration in population sex ratio |

rule in insects. However, the biased population sex ratios that result from the spread of male-killing bacteria can reverse this pattern (31). Male choice of females and competition among females for males is expected, with a relaxation of selection on males to ensure paternity.

Male-killers that have invaded populations may cause changes to host biology. Theory predicts selection for an increase in the size of clutch produced (32). Most importantly, genes that prevent the action or transmission of the parasite will be favored. The presence of these genes has been reported (33), but their nature and mode of action are unknown. The means by which insects exclude bacteria is clearly of great import in our understanding of insect-borne diseases, and the nature of resistance genes is expected to be an important focus of future research.

One of the issues to be determined relates to whether male-killing bacteria can cause the extinction of their host. The case of the butterflies *Acraea encedon* and *A. encedana* is suggestive. The *Wolbachia* male-killer in these species is at high prevalence and clearly has some impact on the host population (21,31). If a male-killing bacterium showed perfect vertical transmission, host extinction would be likely. However, selection on the host acts to lower bacterial transmission efficiency, which may ultimately limit the frequency of extinction.

### Conclusions: Implications and Uses of Male-Killing Bacteria

Male-killing is an adaptive trait that aids the spread of inherited bacteria through natural populations. The presence of male-killing strains in many bacterial taxa clearly indicates that male-killing should be considered in epidemiologic investigations of vertically transmitted bacteria. Male-killing is perhaps most important in interactions between arthropods and *Rickettsia* and *Spiroplasma*. Members of these genera frequently show horizontal transmission between arthropod hosts (after host-feeding), as well as vertical transmission in the arthropod host. Given that some bacteria in these groups induce male-killing, testing for the presence or absence of this trait should be a part of future investigations of their epidemiology.

The potential usefulness of male-killing bacteria in pest control has yet to be properly assessed. Male-killers may be used on their own to reduce host population size. Alternatively, they may be integrated into management schemes based on release of sterile males, so that they may amplify the effect of sterile releases on the population size of adult males. In addition, the recent discovery of male-killing in the clade *Wolbachia* adds an extra dimension to the use of this organism in direct and transgenic control of disease transmission.

The usefulness of male-killers in reducing pest damage on their own is debatable. Insect population size and population persistence are largely a function of female, not male, number. Thus, although the presence of a male-killer may reduce larval density, it is unlikely to decrease the population size of breeding females. Furthermore, the presence of density dependence during the larval stages is likely to reduce the effect of male death on numbers of larvae.

Perhaps a more realistic use of male-killing bacteria in pest management would be in conjunction with sterile male release systems of control. In sterile male release, control is achieved through release into the environment of mass-produced sterile males, which mate with females and lower their fertility (34). The success of sterile male release depends on maintaining a high ratio of sterile to normal males in the population. The presence of a male-killer in the host population lowers the number of fertile males and thus increases the effectiveness of any release. The effects of male-killing bacteria at different prevalences on sterile male release, in conjunction with the effects on host population dynamics, need to be investigated. However, direct use of male-killing bacteria as an aid to controlling host numbers is only achievable as a long-term stratagem. Following release of infected hosts into natural populations, spread will occur only in hosts with suitable ecologies and significant prevalence levels will be achieved over a period of years rather than weeks.

Another potential application of male-killing bacteria in the sphere of pest and disease vector control may occur indirectly through study of the virulence mechanisms of male-killers.

### Acknowledgments

The authors thank Andrew Pomiankowski and two anonymous reviewers for their comments on the manuscript.

In conducting this study, Greg Hurst was supported by a BBSRC D Phillips Fellowship and Frank Jiggins by a BBSRC studentship.

Dr. Hurst has been a BBSRC David Phillips Fellow at University College London since 1997. His research interests center on the dynamics and importance of parasites that affect insect reproduction.

### References

1. Moran N, Baumann P. Phylogenetics of cytoplasmically inherited microorganisms of arthropods. Trends Ecol Evol 1994;9:15-20.
2. Baumann P, Lai C-Y, Baumann L, Rouhbakhsh D, Moran NA, Clark MA. Mutualistic associations of aphids and prokaryotes: biology of the genus *Buchnera*. Appl Environ Microbiol 1995;61:1-7.
3. O'Neill S, Giordano R, Colbert AME, Karr TL, Robertson HM. 16S rRNA phylogenetic analysis of the bacterial endosymbionts associated with cytoplasmic incompatability in insects. Proc Natl Acad Sci U S A 1992;89:2699-702.
4. Hackett KJ, Lynn DE, Williamson DL, Ginsberg AS, Whitcomb RF. Cultivation of the *Drosophila* spiroplasma. Science 1986;232:1253-5.
5. Werren JH, Skinner SW, Huger AM. Male-killing bacteria in a parasitic wasp. Science 1986;231:990-2.
6. Hurst GDD, vd Schulenburg HG, Majerus TMO, Bertrand D, Zakharov IA, Baungaard J, et al. Invasion of one insect species, *Adalia bipunctata*, by two different male-killing bacteria. Insect Molec Biol 1999;8:133-9.
7. Hurst GDD, Hammarton TC, Majerus TMO, Bertrand D, Bandi C, Majerus MEN. Close relationship of the inherited parasite of the ladybird, *Coleomegilla maculata*, to *Blattabacterium*, the beneficial symbiont of the cockroach. Genet Res 1997;70:1-6.
8. Werren JH, Hurst GDD, Zhang W, Breeuwer JAJ, Stouthamer R, Majerus MEN. Rickettsial relative associated with male killing in the ladybird beetle (*Adalia bipunctata*). J Bacteriol 1994;176:388-94.
9. Hurst GDD, Jiggins FM, vd Schulenburg JHG, Bertrand D, West SA, Goriacheva II, et al. Male-killing *Wolbachia* in two species of insect. Proc R Soc Lond B 1999;266:735-40.
10. Whitcomb RF. The genus *Spiroplasma*. Annu Rev Microbiol 1980;34:677-709.
11. Davis MJ, Ying Z, Brunner BR, Pantoja A, Ferwerda FH. Rickettsial relative associated with papaya bunchy top disease. Curr Microbiol 1998;36:80-4.
12. Stouthamer R, Breeuwer JAJ, Hurst GDD. *Wolbachia pipientis*: microbial manipulator of arthropod reproduction. Annu Rev Microbiol 1999;53:71-102.
13. Bandi C, Damiani G, Magrassi L, Grigolo A, Fani R, Sacchi L. Flavobacteria as intracellular symbionts in cockroaches. Proc R Soc Lond B Biol Sci 1994;257:43-8.
14. Jiggins FM, Hurst GDD, Jiggins CD, vd Schulenburg JHG, Majerus MEN. The butterfly *Danaus chrysippus* is infected by a male-killing *Spiroplasma* bacterium. Parasitology 2000;120:439-46.
15. Skinner SW. Son-killer: a third extrachromosomal factor affecting sex ratios in the parasitoid wasp *Nasonia vitripennis*. Genetics 1985;109:745-54.
16. Werren JH. The coevolution of autosomal and cytoplasmic sex ratio factors. J Theor Biol 1987;124:317-34.
17. Hurst GDD, Majerus MEN. Why do maternally inherited microorganisms kill males? Heredity 1993;71:81-95.
18. Roberts LW, Rapmund G, Cadigan FCJ. Sex ratio in *Rickettsia tsutsugamushi* infected and non-infected colonies of *Leptotrombidium* (Acari: Trombiculidae). J Med Entomol 1977;14:89-92.
19. Takahashi M, Urakami H, Yoshida Y, Furuya Y, Misumi H, Hori E, et al. Occurrence of high ratio of males after introduction of minocycline in a colony of *Leptotrombidium fletcheri* infected with *Orientia tsutsugamushi.* Eur J Epidemiol 1997;13:79-86.
20. Williamson DL, Poulson DF. Sex ratio organisms (Spiroplasmas) of *Drosophila*. In: Whitcomb RF, Tully JG, editors. Sex ratio organisms (Spiroplasmas) of *Drosophila*; Vol. III. New York: Academic Press; 1979. pp. 175-208.
21. Jiggins FM, Hurst GDD, Dolman CE, Majerus MEN. High prevalence of male-killing *Wolbachia* in the butterfly *Acraea encedana*. J Evol Biol 2000;13:495-501.
22. Chang KS, Shiraishi T, Nakasuji F, Morimoto N. Abnormal sex ratio condition in the walnut leaf beetle, *Gastrolina depressa* (Coleoptera: Chrysomelidae). Appl Entomol Zool 1991;26:299-306.
23. Counce SJ, Poulson DF. Developmental effects of the sex-ratio agent in embryos of *Drosophila willistoni*. J Exper Zool 1962;151:17-31.
24. Sakaguchi B, Poulson DF. Interspecific transfer of the "sex-ratio" condition from *Drosophila willistoni* to *D. melanogaster*. Genetics 1963;48:841-61.
25. Hurst GDD, Purvis EL, Sloggett JJ, Majerus MEN. The effect of infection with male-killing *Rickettsia* on the demography of female *Adalia bipunctata* L. (two spot ladybird). Heredity 1994;73:309-16.
26. Ikeda H. The cytoplasmically inherited 'sex-ratio' condition in natural and experimental populations of *Drosophila bifasciata*. Genetics 1970;65:311-33.
27. Malogolowkin-Cohen C, Rodriguez-Pereira MAQ. Sexual drive of normal and SR flies of *Drosophila nebulosa*. Evolution 1975;29:579-80.
28. Ebbert M. The interaction phenotype in the *Drosophila willistoni*–spiroplasma symbiosis. Evolution 1991;45:971-88.
29. Sakaguchi B, Poulson DF. Distribution of "sex-ratio" agent in tissues of *Drosophila willistoni*. Genetics 1961;46:1665-76.
30. Hurst GDD, Walker LE, Majerus MEN. Bacterial infections of hemocytes associated with the maternally inherited male-killing trait in British populations of the two spot ladybird, *Adalia bipunctata*. J Invertebr Pathol 1996;68:286-92.
31. Jiggins FM, Hurst GDD, Majerus MEN. Sex ratio distorting *Wolbachia* causes sex role reversal in its butterfly host. Proc R Soc Lond B Bio Sci 2000;267:69-73.
32. Hurst GDD, McVean GAT. Parasitic male-killing bacteria and the evolution of clutch size. Ecol Entomol 1998;23:350-3.

33. Cavalcanti AGL, Falcao DN, Castro LE. "Sex-ratio" in *Drosophila prosaltans*—a character due to interaction between nuclear genes and cytoplasmic factors. Am Nat 1957;91:327-9.
34. Robinson AS. Sex ratio manipulation in relation to insect pest control. Annu Rev Genet 1983;17:191-214.
35. Majerus TMO, Majerus MEN, Knowles B, Wheeler J, Bertrand D, Kuznetsov VN, et al. Extreme variation in the prevalence of inherited male-killing microorganisms between three populations of *Harmonia axyridis* (Coleoptera: Coccinellidae). Heredity 1998;81:683-91.
36. Jiggins FM, Hurst GDD, Majerus MEN. Sex ratio distortion in *Acraea encedon* (Lepidoptera: Nymphalidae) is caused by a male-killing bacterium. Heredity 1998;81:87-91.
37. Hurst GDD, Hammarton TC, Obrycki JJ, Majerus TM, Walker LE, Bertrand D, et al. Male-killing bacteria in a fifth ladybird beetle, *Coleomegilla maculata* (Coleoptera: Coccinellidae). Heredity 1996;77:177-85.
38. Hurst GDD, Bandi C, Sacchi L, Cochrane A, Bertrand D, Karaca I, et al. *Adonia variegata* (Coleoptera: Coccinellidae) bears maternally inherited Flavobacteria that kill males only. Parasitology 1999;118;125-34.
39. Hurst GDD, Majerus MEN, Walker LE. The importance of cytoplasmic male killing elements in natural populations of the two spot ladybird, *Adalia bipunctata* (Linnaeus) (Coleoptera: Coccinellidae). Biol J Linn Soc 1993;49: 195-202.
40. Geier PW, Briese DT, Lewis T. The light brown apple moth *Epiphyas postvittana* (Walker). 2. Uneven sex ratios and a condition contributing to them in the field. Austr J Ecol 1978;3:467-88.
41. Clarke C, Sheppard PM, Scali V. All female broods in the butterfly *Hypolimnas bolina* (L.). Proc Roy Soc Lond B 1975;189:29-37.
42. Brimacombe LC. All-female broods in field and laboratory populations of the Egyptian cotton leafworm, *Spodoptera littoralis* (Boisduval) (Lepidoptera: Noctuidae). Bull Entomol Res 1980;70:475-81.
43. Higashiru Y, Ishihara M, Schaefer PW. Sex ratio distortion and severe inbreeding depression in the gypsy moth *Lymantria dispar* L. in Hokkaido, Japan. Heredity 1999;83:290-7.
